# Distribution characteristics of soil microbial communities and their responses to environmental factors in the sea buckthorn forest in the water-wind erosion crisscross region

**DOI:** 10.3389/fmicb.2022.1098952

**Published:** 2023-01-10

**Authors:** Zhi-Yong Zhang, Fang-Fang Qiang, Guang-Quan Liu, Chang-Hai Liu, Ning Ai

**Affiliations:** ^1^College of Life Science, Yan'an University, Yan'an, Shaanxi, China; ^2^China Institute of Water Resources and Hydropower Research, Beijing, China

**Keywords:** bacterial communities, fungal communities, soil characteristics, meteorological characteristics, sea buckthorn forest, water-wind erosion crisscross region

## Abstract

Soil microorganisms are an important part of forest ecosystems, and their community structure and ecological adaptations are important for explaining soil material cycles in the fragile ecological areas. We used high-throughput sequencing technology to examine the species composition and diversity of soil bacterial and fungal communities in sea buckthorn forests at five sites in the water-wind erosion crisscross in northern Shaanxi (about 400 km long). The results are described as follows: (1) The soil bacterial community of the sea buckthorn forest in the study region was mainly dominated by Actinobacteria, Proteobacteria, and Acidobacteria, and the fungi community was mainly dominated by Ascomycota. (2) The coefficient of variation of alpha diversity of microbial communities was higher in the 0–10 cm soil layer than in the 10–20 cm soil layer. (3) Soil electrical conductivity (36.1%), available phosphorous (AP) (21.0%), available potassium (16.2%), total nitrogen (12.7%), and the meteorological factors average annual maximum temperature (33.3%) and average annual temperature (27.1%) were identified as the main drivers of structural changes in the bacterial community. Available potassium (39.4%), soil organic carbon (21.4%), available nitrogen (AN) (13.8%), and the meteorological factors average annual maximum wind speed (38.0%) and average annual temperature (26.8%) were identified as the main drivers of structural changes in the fungal community. The explanation rate of soil factors on changes in bacterial and fungal communities was 26.6 and 12.0%, respectively, whereas that of meteorological factors on changes in bacterial and fungal communities was 1.22 and 1.17%, respectively. The combined explanation rate of environmental factors (soil and meteorological factors) on bacterial and fungal communities was 72.2 and 86.6%, respectively. The results of the study offer valuable insights into the diversity of soil microbial communities in the water-wind erosion crisscross region and the mechanisms underlying their interaction with environmental factors.

## 1. Introduction

Soil microorganisms are an important part of the forest ecosystem. Their community structure, biological activity, and physiological and ecological adaptations to environmental changes play an important role in the mineralization, humification, and nutrient cycle of soil; the conversion of soil organic compounds and nutrient release; and regulation of the functional diversity of soil (Dong et al., 2014; Liu et al., 2019; Nkongolo and Narendrula-Kotha, 2020). For example, r-selected microorganisms have high cellulose hydrolase activity and an abundance of simple carbohydrate degradation genes, whereas k-selected microorganisms prefer to utilize hard-to-degrade carbon fractions (Fierer et al., 2007). Microorganisms secrete indoleacetic acid analogs and gibberellins, which activate phosphorus and potassium in the soil and promote the mineralization of SOM (Wang et al., 2013; Zhang et al., 2014). Owing to the diversity of microorganisms, complementary, synergistic, redundant, and selective interactions among species can effectively enhance the ecosystem activity and the buffering capacity of soil against external disturbances (Xia et al., 2016). The adaptation of vegetation in arid and semiarid zones heavily relies on the response of soil microorganisms (Saetre and Stark, 2005; Mengual et al., 2014). Soil microorganisms control many processes in the soil ecosystem; therefore, an in-depth understanding of the composition and functional diversity of soil microorganisms is important for elucidating their important role in regulating key ecosystem processes and developing a healthy ecosystem (Griffiths et al., 2004).

The structure and diversity of soil microbial communities are affected by environmental gradients. Soil microorganisms have significant differential sensitivity to many environmental factors such as climate change, soil properties, and plant growth (Li et al., 2016; Wang et al., 2017; Yang et al., 2017; Zhang et al., 2018). Ecological factors exert additive and interactive effects on soil microorganisms by altering their nutrient requirements, community structure, and energy channels. Garcia-Pichel et al. (2013) reported that the distribution of the cyanobacterium Microcoleus vaginatus is mainly influenced by the temperature at the intercontinental scale. Xia et al. (2016) reported that the diversity of soil bacterial communities in typical forests in China was parabolic along the latitudinal belt and had regional similarities. Yuan et al. (2014) reported that soil bacterial communities at the soil depth of 1–5 cm in the south-facing slope of Nyainqentanglha Mountains, Central Tibetan Plateau, Qinghai-Tibet Plateau, were mainly influenced by precipitation and soil NH_4_^+^ concentration, whereas those at the soil depth of 5–20 cm are mainly influenced by pH. Differences among ecosystems and spatial scales are influenced by environmental factors and result in different geographic distribution patterns of soil microorganisms (Bardgett and van der Putten, 2014; Hu et al., 2019). Therefore, important environmental factors that influence the mechanisms underlying microbial diversity and maintenance in an ecosystem should be identified for examining the functions of soil microorganisms in the ecosystem and reducing uncertainties associated with ecosystem models.

The water-wind erosion crisscross region is located in the center of the Loess Plateau. The ecological environment of this region is fragile, and re-vegetation is the main way to restore the local ecosystem (Chen et al., 2021). After years of ecological engineering and vegetation restoration and reconstruction, the sea buckthorn forest has become one of the most widely distributed and used tree species in the ecological construction of the region. To date, studies have extensively reported on sea buckthorn forests in this region, mainly focusing on soil moisture characteristics (Jian et al., 2015; Gou and Zhu, 2021), soil and water conservation (Wu and Zhao, 2000), and soil quality (Cao et al., 2007; Zhang and Chen, 2007). Most studies had focused on small watersheds; however, little is known regarding the spatial heterogeneity of the composition of soil microbial communities in sea buckthorn forests at the scale of this sample zone. The study of the structure and diversity of soil microbial communities in this sample zone is beneficial for understanding the functions of aquatic ecosystems, wind erosion in ecotones, and the interaction between the environment and microorganisms. Therefore, we selected the water-wind erosion crisscross region in northern Shaanxi as the study region and the sea buckthorn forest in the study region as the study object to examine the structure and diversity of soil microbial communities in the forest and elucidate the relationship among soil, meteorological factors, and soil microorganisms. This study provides a theoretical basis for understanding the biodiversity protection in this region and a scientific basis and data support for promoting research into the ecological restoration effects and sustainable management of sea buckthorn plantations.

## 2. Materials and methods

### 2.1. Study area

The water-wind erosion crisscross region in northern Shaanxi was selected as the study region. The terrain is high in the northwest and low in the southeast. The basic landform types are loess tableland, ridge, hillock, and gully. The region has a semiarid temperate continental monsoon climate and four seasons and receives plenty of sunshine. The climate is changeable, the temperature difference is large, the precipitation is less, the distribution is not uniform, spring is windy and sandy, summer is rainy, and winter is cold and dry. The main soil types in the study region include loess and eolian sandy soils, which are weakly alkaline (pH ≈ 8.0). At present, vegetation in the study region is dominated by *Robinia pseudoacacia* L., *Populus simonii* Carr, *Hippophae rhamnoides* Linn., *Caragana korshinskii* Kom., *Amorpha fruticosa* Linn., *Artemisia gmelinii*, and other artificial vegetation communities.

### 2.2. Plot design and soil sampling

**Figure 1:**
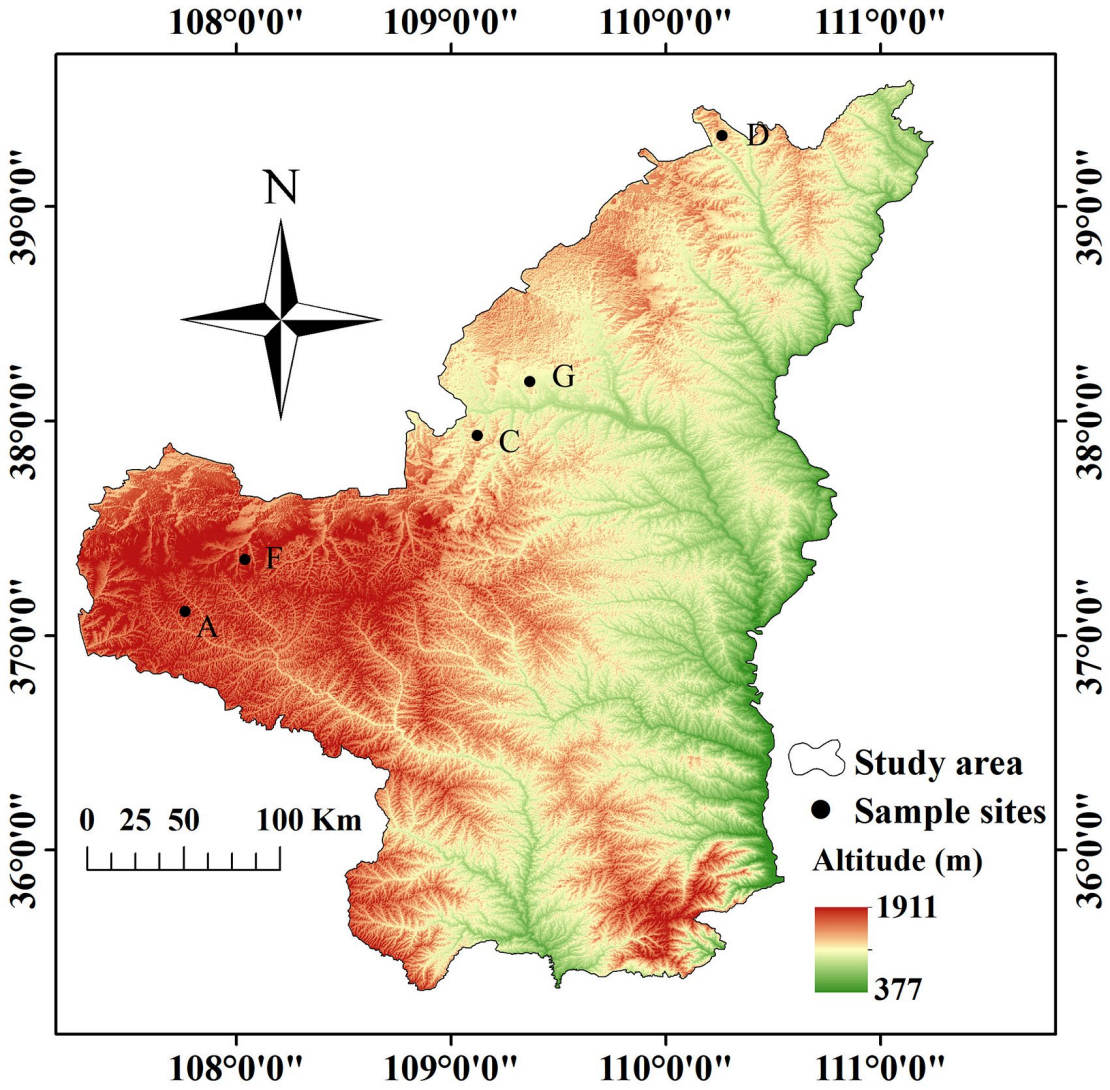
Schematic diagram of the study region.

Based on preliminary forestry data and field survey, the water-wind erosion crisscross region (sample zone length of approximately 400 km) in Wuqi County (Wangwazi and Changcheng towns), Hengshan District, Yuyang District, and Shenmu City in northern Shaanxi Province was selected as the study region (Figure 1). Sea buckthorn plantations with similar site conditions and the same planting years were selected as study objects at each location. We set up three independent plots of 10 m × 10 m in the sea buckthorn forest area at each site. Plot information was recorded (Table 1), and samples were collected according to the five-point sampling method. The depth of soil sampling section was 20 cm. Surface litter was removed, each soil profile was divided into 2 layers vertically downward from the surface, i.e., 0–10 cm and 10–20 cm. A ring knife was used for sampling, and three samples were collected from each layer of the same section for determining the physical properties of the soil. Soil drills (disinfected with 75.0% alcohol before use) were used to collect soil samples. Soil samples from all sampling points of each plot were merged into one soil sample. Roots and stones were removed, and samples were divided into two parts as follows: one part was naturally air-dried and used for examining the physicochemical properties of soil, whereas the other part was stored at −80°C for microbial DNA analysis.

**Table 1:** Basic information of the study region.

Sample area	Sample code	Altitude (m)	Aspect	Slope (°)	Stem base diameter (cm)	Height (m)	Understory herbaceous species
Wuqi county (Wangwazi)	A	1,566	Shady slope	13	2.9	1.2	*Artemisia frigida* Willd, *Artemisia gmelinii*, and *Leymus secalinus* (Georgi) Tzvel
Wuqi county(Changcheng)	F	1,565	Shady slope	20	3.2	1.5	*Bothriochloa ischaemum* (L.) Keng, *Artemisia gmelinii*, *Artemisia sacrorum* Ledeb, and
Hengshan District	C	1,212	Shady slope	12	1.3	0.9	*Setaria viridis* (L.) Beauv *Heteropappus hispidus* (Thunb.) Less and *Astragalus melilotoides* Pall
Yuyang district	G	1,293	Shady slope	6	1.6	0.8	*Astragalus adsurgens* Pall, *Aster hispidus* Thunb, and *Artemisia desertorum* Spreng
Shenmu city	D	1,238	Shady slope	3	2.6	1.2	*Artemisia desertorum* Spreng, Lespedeza bicolor Turcz, *Astragalus melilotoides* Pall, and *Aster hispidus* Thunb

### 2.3. Determination of samples

#### 2.3.1. Soil analysis

Bulk density (BD), total soil porosity (TCP), and capillary water-holding capacity (CWHC) were determined using standard methods (Institute of Soil Science; Chinese Academy of Sciences, 1978). The pH and electrical conductivity (EC) of soil were measured using a PHS-320 high-precision intelligent pH meter (soil and water ratio of 2.5:1) and a DDS-608 multifunctional conductivity meter (soil and water ratio of 5:1), respectively. The concentration of soil organic carbon (SOC) was determined using the dichromate oxidation method. The concentration of available nitrogen (AN) was determined using the alkaline hydrolysis-diffusion absorption method. The concentration of ammonium (NH_4_^+^) and nitrate (NO_3_^−^) was determined using the KCL (2 mol/L) extraction method. The concentration of available potassium (AK) was determined *via* extraction with NH_4_OAc and flame photometry. The concentration of available phosphorous (AP) was determined *via* extraction with 0.5-mol/L NaHCO_3_ and silica-molybdenum blue colorimetry. The concentration of total nitrogen (TN) and total phosphorus (TP) was determined using an automatic discontinuous chemical analyzer (CleverChem Anna, Germany; Bao, 2000).

#### 2.3.2. Determination of microbial samples

Soil microbial samples were examined by Shanghai Personal Biotechnology Co., Ltd.^1^  The experimental process mainly included the following six aspects: (1) Total DNA extraction from the microbiome, DNA quantification (NanoDrop), and separation of DNA fragments *via* agarose gel electrophoresis (1.20% gels). (2) PCR amplification of target fragments using target sequences such as microbial ribosomal RNA or specific gene fragments that can reflect the composition and diversity of the target flora: Primers corresponding to the conserved regions in sequences were designed, sample-specific barcode sequences were added, and variable regions (single or consecutive multiple) of rRNA genes or specific gene fragments were amplified *via* PCR. (3) Recovery of amplification products *via* magnetic bead purification: Approximately 25 μl of the PCR product was mixed with 0.8 times the volume of magnetic beads (Vazyme VAHTSTM DNA Clean Beads). The mixture was shaken to suspend the product and allowed to adsorb on magnetic beads on a magnetic rack for 5 min. The supernatant was aspirated with a pipette gun, and 20 μl of 0.8 times the volume of a magnetic bead washing solution was added to the sample. The sample was shaken and allowed to adsorb on magnetic beads on a magnetic rack for 5 min. The supernatant was removed, and 200 μl of 80% ethanol was added. Adsorption was reversed on the magnetic rack, and the sample on the other side of the PCR tube was allowed to adsorb on magnetic beads. The supernatant was removed, and the sample was incubated at room temperature for 5 min to allow the alcohol to evaporate completely. After cracks were observed on the magnetic beads, 25 μl of the elution buffer was added to the sample, and the PCR tube was placed on the adsorption rack for 5 min until the sample was completely adsorbed. The supernatant was collected in a clean 1.5 mL centrifuge tube and stored until further use. (4) Quantification of the recovered amplification products *via* fluorescence analysis: The Quant-iT PicoGreen dsDNA Assay Kit and a microplate reader (BioTek, FLx800) were used for analysis. Based on the results of fluorescence quantification, each sample was mixed in the corresponding ratio according to the sequencing volume requirement of each sample. (5) Sequencing library preparation: The TruSeq Nano DNA LT Library Prep Kit (Illumina) was used to construct a sequencing library. (6) High-throughput sequencing: Libraries were checked on an Agilent Bioanalyzer using the Agilent High Sensitivity DNA Kit before sequencing. The libraries were quantified on a QuantiFluor fluorescence quantification system, and the qualified libraries (index sequences were not reproducible) were diluted in a gradient, mixed in the appropriate ratio according to the required sequencing volume, and denatured to single strands using NaOH.

The bacterial PCR primers were included 338F (‘ACTCCTACGGGAGGCAGCA’) and 806R (‘GGACTACHVGGGTWTCTAAT’), and the fungal PCR primers included ITS5F (‘GGAAGTAAAAGTCGTAACAAGG’) and ITS1R (‘GCTGCGTTCTTCATCGATGC’).

#### 2.3.3. Meteorological data

The following six meteorological factors were examined: average annual temperature (AAT), average annual maximum temperature (AAMaxT), average annual minimum temperature (AAMinT), average annual precipitation (AAP), average annual sunshine hours (AARSHs), average annual relative humidity (AARH), average annual wind speed (AWS), and average annual maximum wind speed (MAX-WS). The data were extracted from the China Meteorological Data Network^2^ from 1970 to 2016.

### 2.4. Data processing and statistical analysis

The Excel 2016 software was used to preprocess data. The SPSS Statistics (version 22.0) software was used for one-way analysis of variance. One-way analysis of variance was used to compare the physical and chemical properties of soil among different groups (significant differences were determined at 95% confidence intervals). When significance was detected (*p* < 0.05), Duncan’s method was used for multiple comparisons. QIIME 2 (2019.4) was used to process the amplicon sequencing data, and DADA2 (Divisive amplicon denoising algorithm; Callahan et al., 2016) was used to perform quality control, denoising, splicing, and dechimerization of the original sequencing sequence to obtain the amplified sequence variant Amplicon sequence variant (ASV). The classify-sklearn (Bokulich et al., 2018) algorithm in feature-classifier (*via* q2-feature-classifier) was used to annotate a series of amplified sequence variants, and the unleveled ASV/OTU table was used to calculate the microbial α diversity index by calling the “qiime diversity alpha-rarefaction” command. The Canoco 5.0 software was used to perform redundancy analysis (RDA) between bacterial and fungal communities with a relative abundance of >1% and environmental factors. Variance partitioning analysis (VPA) was performed using the “Vegan” package of the R Software, and the Origin 2018 and ArcGis 10.3 software were used for mapping.

## 3. Results

### 3.1. Microecological environmental characteristics of sea buckthorn

#### 3.1.1. Characteristics of soil properties

**Figure 2:**
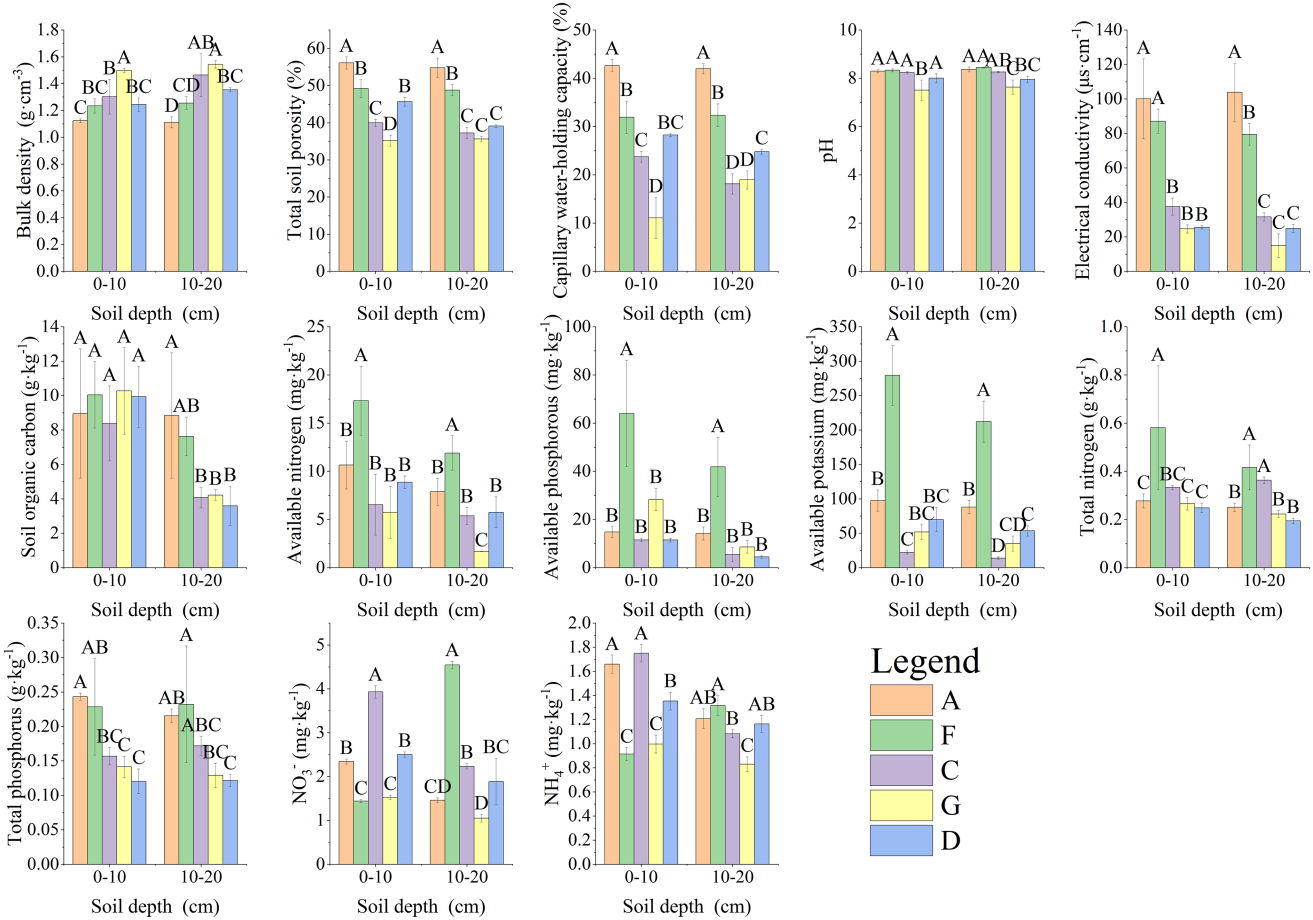
Different soil index characteristics. Different capital letters in the figure indicate that soil indices of the same soil layer differ significantly among different sites (*p* < 0.05).

The soil collected from the study region was weakly alkaline, with pH values ranging from 7.51 to 8.44. pH values were higher in samples A and F. The physical and chemical properties of soil collected from different areas were significantly different (*p* < 0.05). TCP, CWHC, EC, and OC were highest in sample A, whereas the concentration of AN, AP, AK, and TN was highest in sample F. The soil texture of samples C, G, and D was poor, and most soil index values of various plots decreased with an increase in soil depth (Figure 2).

#### 3.1.2. Characteristics of meteorological factors

**Figure 3:**
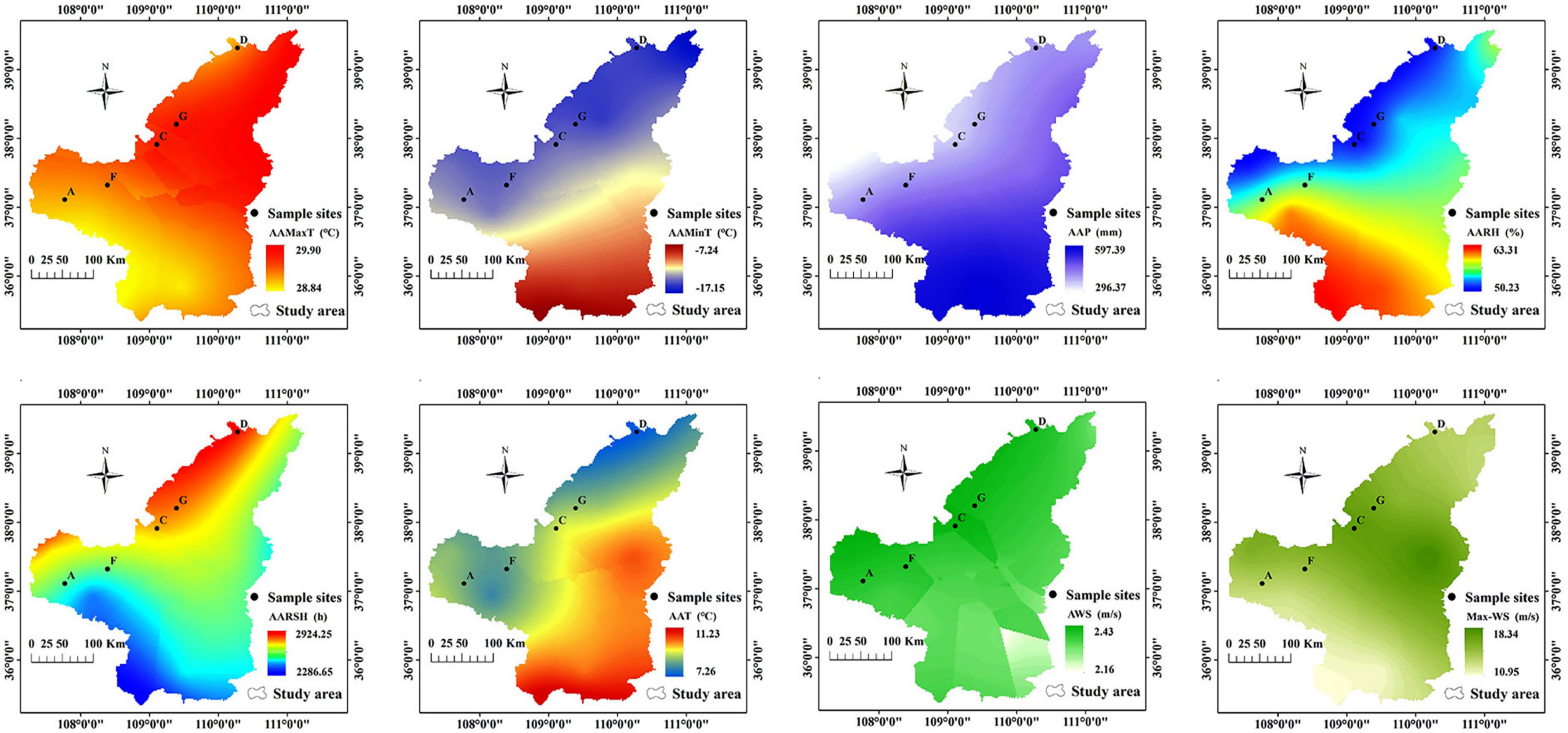
Meteorological distribution map of northern Shaanxi.

In the study region, AAP ranged from 367 to 405 mm; AARH ranged from 52.0 to 56.0%; AARSHs were > 2,500 h; and AAT, AAMaxT, and AAMinT were approximately 8.60°C, 29.0°C, and −14.0°C, respectively. AWS was approximately 2.30 m/s, and Max-WS ranged from 13.7 m/s to 16.8 m/s. Altogether, differences in AAP and Max-WS were significant among different locations (*p* < 0.05; Figure 3).

### 3.2. Characteristics of alpha diversity of soil microbial communities

#### 3.2.1. Characteristics of alpha diversity of soil bacterial communities

**Figure 4:**
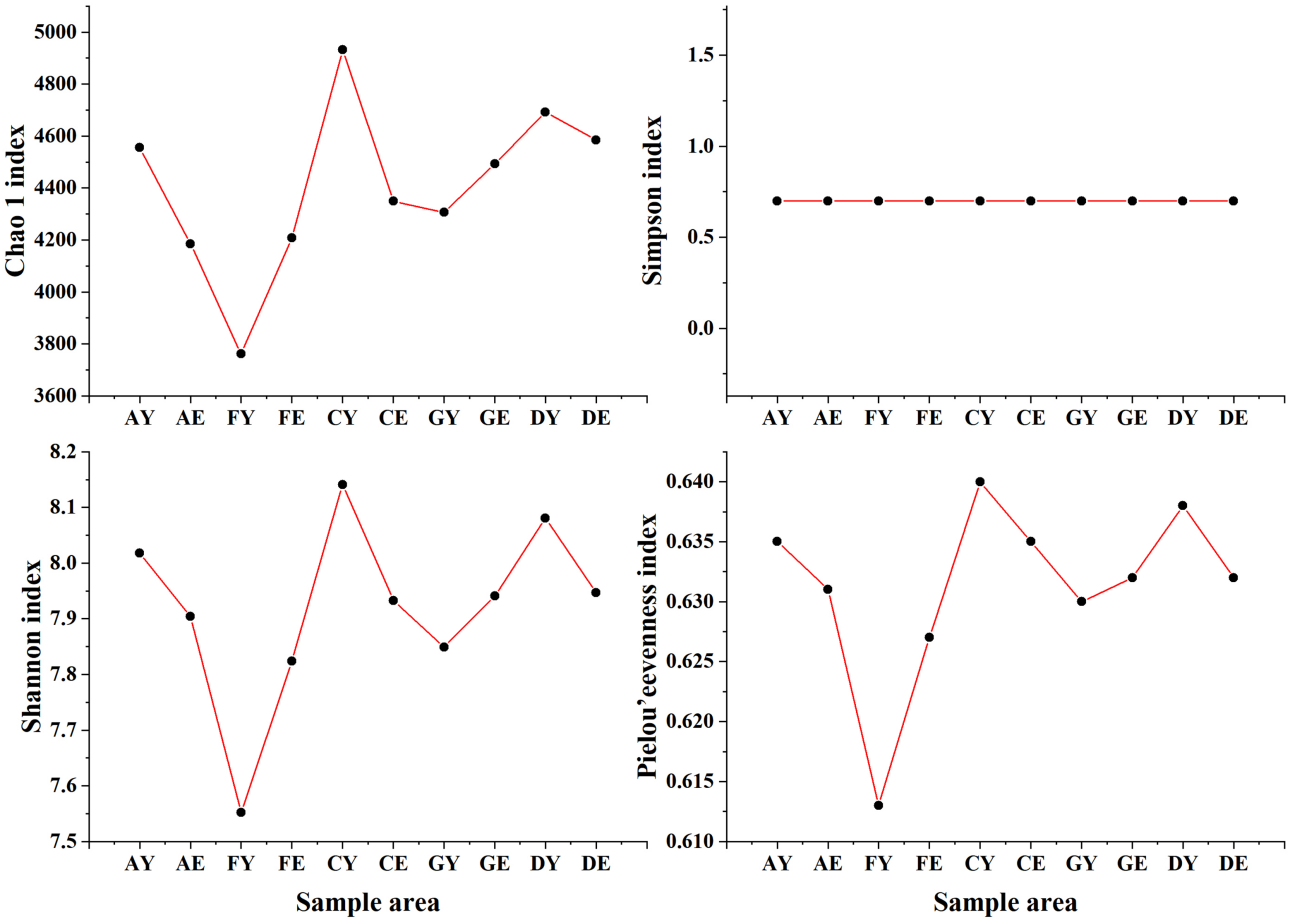
Alpha diversity index of bacterial communities. AY, FY, CY, GY, and DY represent the 0–10 cm soil layer of plots A, F, C, G, and D, respectively; AE, FE, CE, GE, and DE represent the 10–20 cm soil layer of plots A, F, C, G, and D, respectively.

At a soil depth of 0–10 cm, the Chao1 and Shannon indices of soil bacterial communities at different sites were different (C > D > A > G > F), and the Pielou’s evenness index ranged from 0.613 to 0.638 across different sites. At a soil depth of 10–20 cm, the Chao1 and Shannon indices of soil bacterial communities at different sites ranged from 4184.84 to 4584.65 and 7.82 to 7.95, respectively, with the index values being higher in samples D and G and lower in samples A and F (Figure 4).

The Simpson index of soil bacterial communities did not change with an increase in soil depth and was approximately 0.699. The Chao1, Shannon, and Pielou’s evenness indices were higher in samples A, C, and D collected at a soil depth of 0–10 cm than in those collected at a soil depth of 10–20 cm. However, an opposite trend was observed for samples F and G. The coefficients of variation of the Chao1, Shannon, and Pielou’s evenness indices were higher among samples collected at a soil depth of 0–10 cm than among those collected at a soil depth of 10–20 cm (Figure 4).

#### 3.2.2. Characteristics of alpha diversity of soil fungal communities

**Figure 5:**
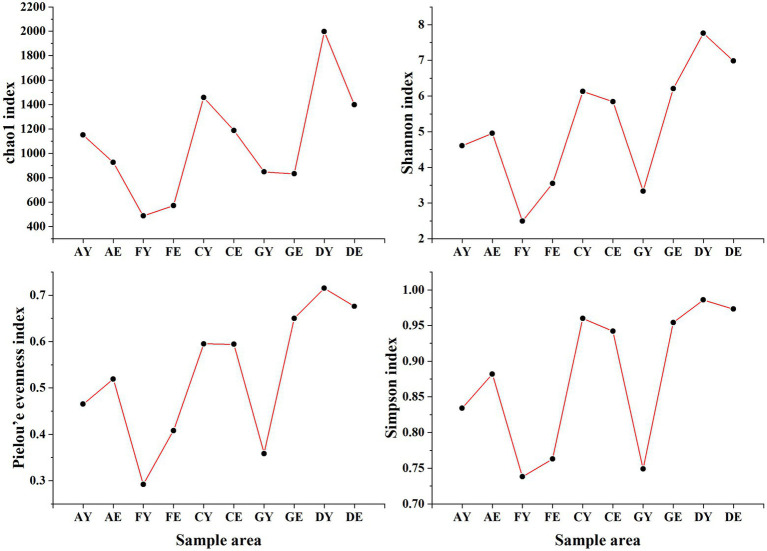
Alpha diversity index of fungal communities. AY, FY, CY, GY, and DY represent the 0–10 cm soil layer of plots A, F, C, G, and D, respectively; AE, FE, CE, GE, and DE represent the 10–20 cm soil layer of plots A, F, C, G, and D, respectively.

The alpha diversity indices of soil fungal communities were different at the same site. The Chao1, Simpson, Shannon, and Pielou’s evenness indices were highest in sample D and lowest in sample F at a soil depth of 0–10 cm and 10–20 cm. Except the Chao1 index, all index values increased with an increase in depth in samples A and G. However, the alpha diversity indices of samples C, D, and F decreased with an increase in depth. The coefficients of variation of the Chao1, Shannon, and Pielou’s indices were higher in soil samples collected at a depth of 0–10 cm than in those collected at a depth of 10–20 cm (Figure 5).

### 3.3. Structural composition of soil microbial communities

#### 3.3.1. Structural composition of soil bacterial communities

**Figure 6:**
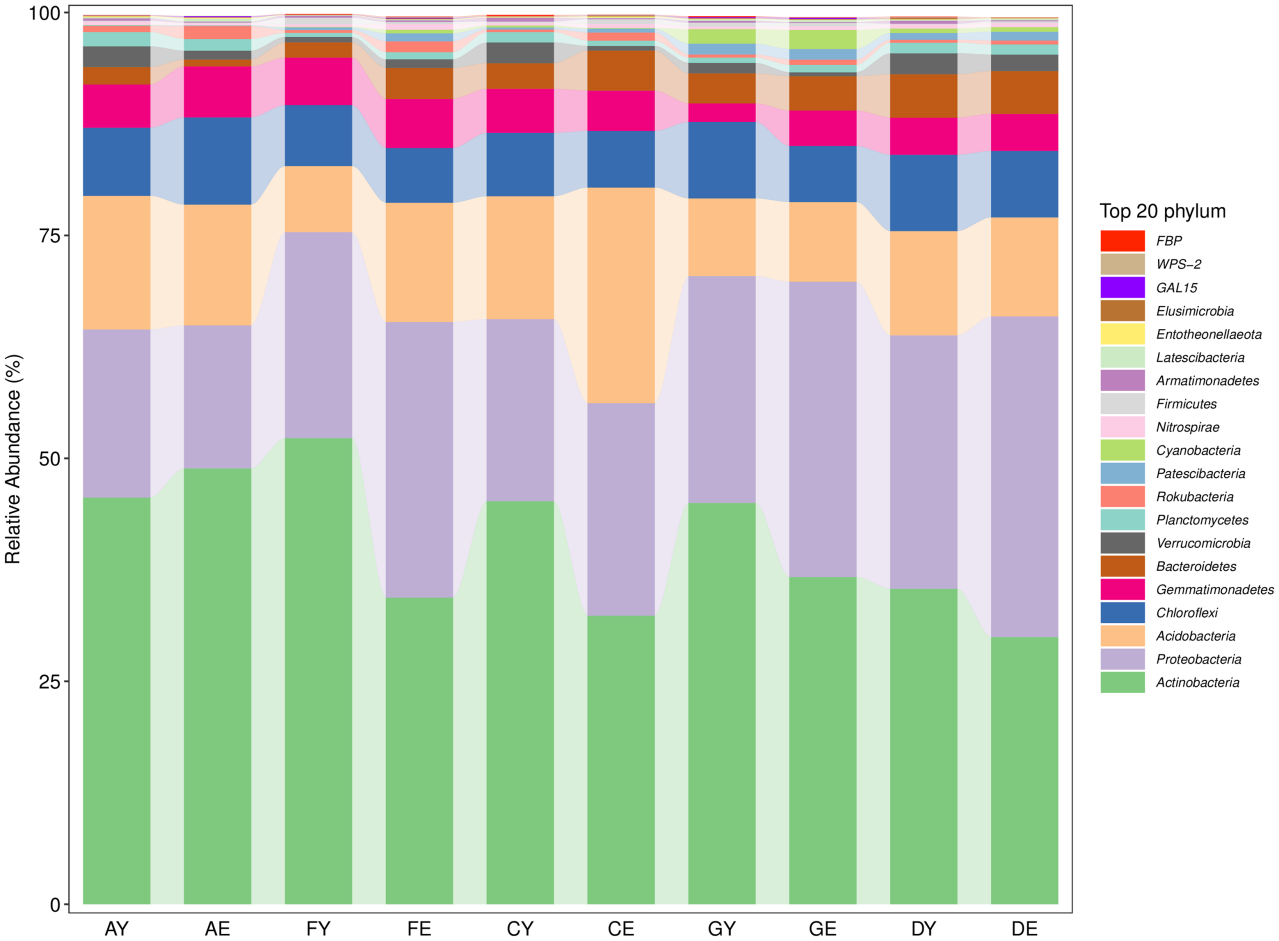
Horizontal composition of soil bacterial phyla.

The bacterial sequences read from each soil sample belonged to 36 phyla (Supplementary Table 2). At the phylum level, soil bacterial taxa in different locations were mainly dominated by Actinobacteria, Proteobacteria, and Acidobacteria, and the relative abundance of these bacterial groups was >10%. The relative abundance of Chloroflexi, Gemmatimonadetes, Bacteroidetes, and Verrucomicrobiota was >1%. The relative abundance of other bacterial groups was <1%, indicating that they were rare. Actinobacteria was the most dominant phylum at each site, and the composition of dominant bacterial communities (phylum level) was similar among different sites (Figure 6).

#### 3.3.2. Structural composition of soil fungal communities

**Figure 7:**
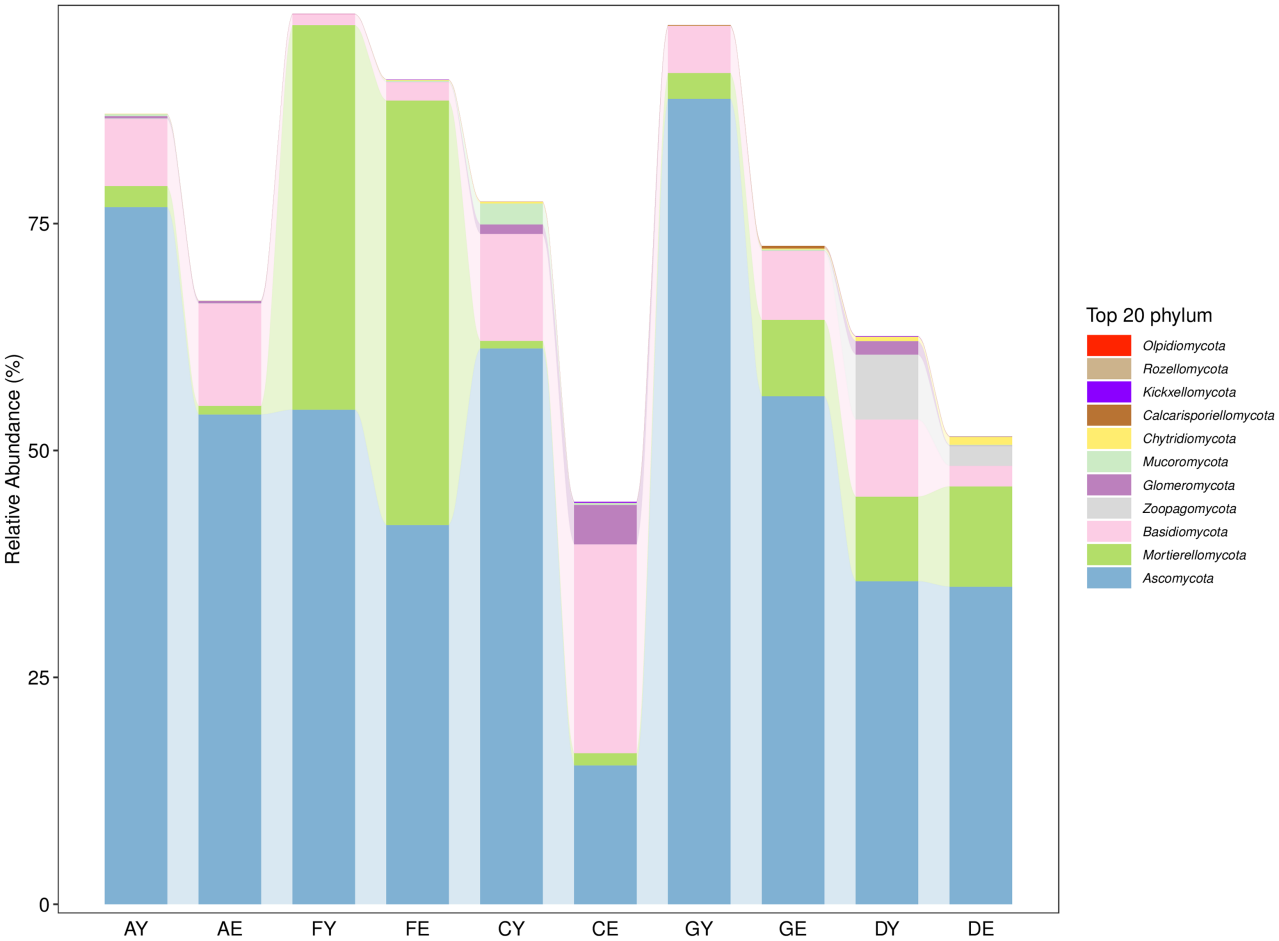
Horizontal composition of soil fungal phyla.

The fungal sequences read from each soil sample belonged to 11 phyla (Supplementary Table 3). At the phylum level, the relative abundance of Ascomycota, Mortierellomycota, and Basidiomycota was >1%. Ascomycota was the most dominant phylum, whereas the relative abundance of other fungal groups was <1% (Figure 7).

### 3.4. Correlation between soil microbial communities and environmental factors

#### 3.4.1. Correlation between bacterial communities and environmental factors

**Figure 8:**
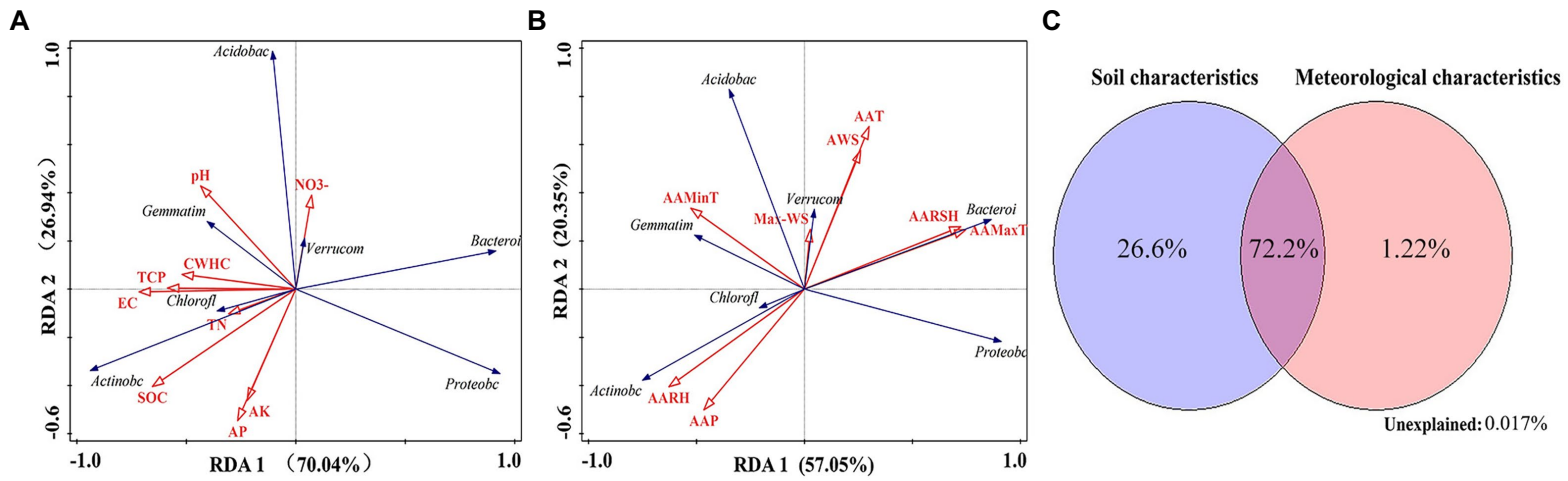
**(A)** Redundancy analysis of bacterial communities (phylum level) and soil factors. **(B)** Redundancy analysis of bacterial communities (phylum level) and meteorological factors. **(C)** Variation partitioning analysis of the distribution of bacterial communities (phylum level) explained by microecological environmental factors.

The results of RDA showed that the soil-related factors EC (36.1%), AP (21.0%), AK (16.2%), and TN (12.7%) and the meteorological factors AAMaxT (33.3%) and AAT (27.1%) had a higher impact on changes in the structure of dominant bacterial communities at the phylum level (Monte Carlo replacement test, *p* < 0.01; Figures 8A,B). The Actinobacteria phylum was positively correlated with SOC, TN, AARH, and AAP. The Acidobacteria and Verrucomicrobia phyla were positively correlated with NO^−^_3_ and Max-WS. The Chloroflexi phylum was positively correlated with TN, EC, and AARH. The Gemmatimonadetes phylum was positively correlated with pH and AAMinT. The Proteobacteria phylum was negatively correlated with CWHC, TCP, pH, and AAMinT. The Bacteroidetes phylum was positively correlated with AARSH and AAMaxT (Figures 8A,B).

The results of VPA showed that soil-related and meteorological factors accounted for 26.6 and 1.22% of variations in the structure of soil bacterial communities, respectively. When combined, these factors accounted for 72.2% of variations in the structure of soil bacterial communities (phylum level; Figure 8C).

#### 3.4.2. Correlation between fungal communities and environmental factors

**Figure 9:**
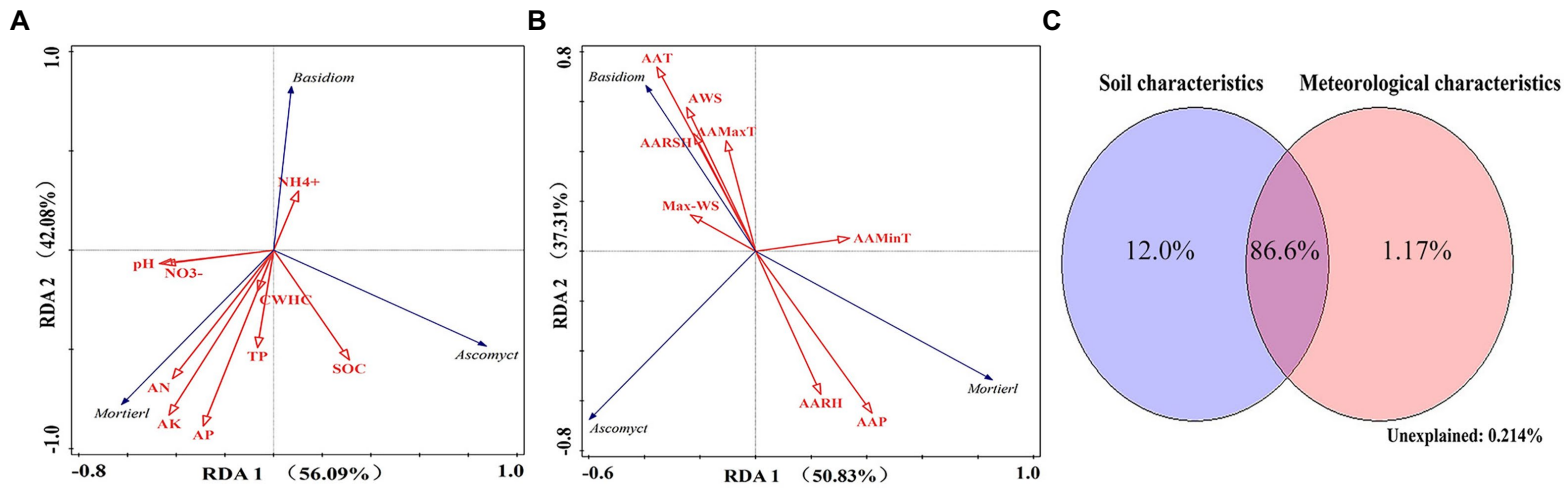
**(A)** Redundancy analysis of fungal communities (phylum level) and soil factors. **(B)** Redundancy analysis of fungal communities (phylum level) and meteorological factors. **(C)** Variation partitioning analysis of the distribution of fungal communities (phylum level) explained by microecological environmental factors.

The results of RDA showed that the soil factors AK (39.4%), SOC (21.4%), and AN (13.8%) and the meteorological factors Max-WS (38.0%) and AAT (26.8%) had a higher impact on changes in the structure of dominant fungal communities at the phylum level (Monte Carlo permutation test, *p* < 0.01; Figures 9A,B). The Mortierellomycota phylum was positively correlated with AK, AN, and AAP. The Basidiomycota phylum was positively correlated with NH_4_^+^, AAT, AWS, AAMaxT, and AARSH and negatively correlated with AAP and AARH. The Ascomycota phylum was positively correlated with SOC and negatively correlated with AAMinT (Figures 9A,B).

The results of VPA showed that soil and meteorological factors accounted for 12.0 and 1.17% of variations in the fungal community structure, respectively. When combined, these factors accounted for 86.6% of variations in the fungal community structure (phylum level; Figure 9C).

## 4. Discussion

### 4.1. Analysis of the structure and diversity of soil microbial communities

Soil bacteria and fungi can rapidly and sensitively respond to environmental changes, and the evolution of their community (composition, quantity, and diversity) is an important indicator of their ecological function and the evolution of soil environmental quality. In this study, a total of 36 bacterial communities and 11 fungal communities (Figures 6, 7) were found in soil collected from different parts of the sea buckthorn forest in the study region. In the study region, dominant microbial communities were the same at different sites. The soil bacterial community was dominated by Actinobacteria, Proteobacteria, and Acidobacteria, whereas the fungal community was dominated by Ascomycota. These results are consistent with those of previous studies. These microbial communities are widely distributed in Qinling Mountains (Zhao et al., 2022), Xinjiang (Liu et al., 2020), the Qinghai-Tibet Plateau (Wang et al., 2020), Karst regions (Hu et al., 2020), South Africa (Gqozo et al., 2020), and the Arctic (Grau et al., 2017).

The ability of dominant microbial communities to tolerate ecological stress and promptly respond to resource pulses helps them to survive in arid ecological environments. Studies have shown that ecological amplitude largely controls the community structure and composition of soil microorganisms. Actinomycetes are highly tolerant and resistant to drought, heat, and radiation, as they produce spores and grow in a filamentous form, with high dispersal capacity. In addition, they are the main functional bacteria for degrading lignin and cellulose (Bhatti et al., 2017). Acidobacteria are involved in various soil ecological processes and play an important role in nitrate, nitrite, carbon monoxide, and iron redox cycling in soil. They can use different carbon substrates and degrade complex substrates such as xylan and hemicellulose (He et al., 2009), and their photosynthetic and oligotrophic characteristics help them to survive in extreme environments such as drought and contaminated environments. Sul et al. (2013) reported that Acidophilus and Actinomycetes can survive in habitats with very low organic carbon content. Proteobacteria are the most abundant phyla in the bacterial domain, and Proteobacteria are the main functional bacteria for the transformation of organic matter. Proteobacteria include α-Proteobacteria, β-Proteobacteria, and δ-Proteobacteria and can decompose organic matter to produce nutrients such as ammonia and methane during nitrogen, phosphorus, and other nutrient cycles. Maintenance of soil fertility is important (Dedysh et al., 2004; Lv et al., 2014). Egidi et al. (2019) reported that fungi such as Ascomycota dominate soil fungal communities in most regions of the world and have a wide ecological niche. Ascomycota can decompose plant and animal residues and release bioavailable nutrient elements to promote soil nutrient cycling; improve soil quality; and enhance the colonization, growth, and development of desert plants (Zhou et al., 2021). Sea buckthorn bushes can effectively aggregate soil microorganisms and provide a living space for microbial survival. In addition, the diverse lifestyles of saprophytic, symbiotic, and parasitic fungi provide good stress resistance for surviving in arid and fragile environments. The arid climate and the high intensity of solar radiation in this study area led to the special ecological environment such as poor soil. In addition, the particularity of activities such as the living and reproductive mode of dominant bacteria makes the microorganisms and the environment show a good relationship.

### 4.2. Analysis of the relationship between microbial communities and environmental factors

The microbial community niche is defined by the interaction between environmental factors such as soil-related and meteorological factors, which, in turn, affects the structural composition and diversity of the microbial community. In this study, soil-related and meteorological factors were found to have a significant impact on changes in the composition of microbial communities (relative abundance of >1%) to a greater extent. However, compared with soil-related factors, meteorological factors had a lower impact on fungal and bacterial communities, which may be because the climate of the study region was the same throughout the study, and the spatial heterogeneity of meteorological factors among different sites was low. Zeng et al. (2019) reported that soil-related and meteorological factors highly influence the changes in bacterial communities. However, when they analyzed factors driving the changes in bacterial communities along multiple environmental gradients in the east–west 800 km transect of the Loess Plateau, they found that soil-related factors had a higher impact on bacterial communities. Another study revealed that meteorological factors control the geographical and spatial distribution patterns of microorganisms in a large-scale space. Zheng et al. (2020) found that the beta diversity of bacterial communities in China is significantly associated with the climatic zone pattern from 21.6°N to 50.8°N, and the annual average temperature and annual precipitation are important factors affecting the beta diversity of soil bacteria. Yang and Wu (2020) reported that the Shannon and Simpson indices of soil bacteria in subtropical and cold temperate zones were higher than those in warm temperate and mid-temperate zones.

In this study, soil-related factors such as EC, AP, AK, and TN and meteorological factors such as AAMaxT and AAT had a higher impact on changes in the structure of bacterial communities (relative abundance of >1%; Figure 8). In addition, AK, OC, AN, Max-WS, and AAT had a greater impact on changes in the structure of fungal communities (Figure 9). In the water-wind erosion crisscross region, environmental factors such as soil and meteorological characteristics control the structure of microbial communities through selection or determination (Raes and Bork, 2008). The soil in the study region has been in an alkaline environment for a long time. Soil EC reflects the clay, salt content, and mineral content of the soil to a certain extent; characterizes living conditions such as water and air required for the survival of microorganisms (Torsvik and Øvreås, 2002); and affects the distribution and activity of microorganisms in the soil. Negassa et al. (2015) found that cellulolytic bacteria such as Proteobacteria, Actinomycetes, and Firmicutes mainly exist in large pores, whereas Acidobacteria exist in small pores. The activity of microorganisms can be altered by altering the soil pore structure, which affects the microenvironment of their survival (Helliwell et al., 2014). The degree of soil salinization has potential negative effects on the composition and diversity of soil bacterial communities (Uddin et al., 2019). Vegetation provides carbon, nitrogen, and other substances to the soil through litter and root exudates. The spatial distribution of soil carbon, nitrogen, phosphorus, potassium, and other elements in different soil layers is an important reason for variations in microbial diversity. The availability of soil nutrients is an important limiting factor for the activity and composition of microbial communities (Chen et al., 2015; Zhao et al., 2016). Phosphorus can limit the utilization of carbon by microorganisms (Demoling et al., 2007; Esberg et al., 2010) and alter the abundance and structure of soil bacterial communities (Estrada-Bonilla et al., 2017). In addition, phosphorus-solubilizing microbes in the soil can secrete organic acids, phosphatases, and other substances; promote the activation of phosphorus in soil; alter the soil phosphorus form; and improve the availability of phosphorus (Yousefi et al., 2011; Srinivasan et al., 2012; Li et al., 2020). Liu et al. (2020b) and Wei et al. (2016) found that phosphate-solubilizing bacteria can decompose residual phosphorus and HCl-Pi in soil, thus increasing the content of phosphorus (NaOH-Pi and NaOH-Po) in soil. Nitrogen and potassium have significant effects on bacterial and fungal communities. Deng et al. (2020) reported that total carbon, TN, TP, AP, AN, and AK in soil were important factors affecting the microbial community in different types of vegetation in open-pit iron ore mining areas. Liu et al. (2020a) found that BD, soluble carbon, TN, and plant abundance had a greater impact on the microbial community during long-term secondary succession. Liu et al. (2015) reported that the carbon content of soil drives the biogeographical distribution of fungal communities in the black soil region of Northeast China. These results are consistent with those of the present study. In addition, although the impact of SOC on the overall changes in bacterial communities in this study was low, related studies have shown that SOC provides energy and carbon for microbial activity. In this study, SOC was positively correlated with Actinomycetes. Proteobacteria and Actinomycetes are correlated with dissimilatory nitrate reduction, denitrification, and nitrification pathways in soil and act as the main CO_2_ fixers, thus playing an important role in promoting soil carbon and nitrogen cycling and improving the stability of the ecosystem. You et al. (2014) reported that the relative abundance of Gram-negative bacteria, saprophytic fungi, and Actinomycetes is significantly associated with variations in the specific activity of soil enzymes involved in SOC transformation or turnover.

Owing to global climate change, soil microbial communities may passively respond to climate changes such as global warming, resulting in community succession differentiation (Guo et al., 2018). In this study, wind speed and temperature had significant effects on changes in microbial communities (Figures 8, 9). Wind erosion is the main natural force of soil erosion in the water-wind erosion crisscross region and can affect the living space of microbial communities through weathering. The stronger temperature variability in the study region results in higher physiological stress on microorganisms (Van Gestel et al., 2011), affecting their metabolic rate and diversity (Hendershot et al., 2017). Active organic carbon can be rapidly decomposed by microorganisms under warm conditions (Allison et al., 2010). Exogenous carbon input can increase the abundance of soil microorganisms, and increased temperature can accelerate the respiration rate and accumulation of soil microorganisms. Studies have suggested that global warming may increase nutrient competition between plant roots and microorganisms by promoting crop growth, favoring nutrient uptake by plants, and leading to a scarcity of nutrients required for the survival of soil microbes, thus limiting microbial growth (Hu et al., 2001). Frindte et al. (2019) examined soil microbial communities in the Arctic-alpine region and found that temperature and moisture were the driving factors controlling changes in local microbial communities. In addition, Shi et al. (2020) reported that interannual fluctuations in the mean annual precipitation and temperature were significantly associated with age-related changes in the structure of soil bacterial and fungal communities in the Tibetan Plateau.

## 5. Conclusion

The bacterial community is dominated by Actinobacteria, Proteobacteria, and Acidobacteria, whereas the fungal community is dominated by Ascomycota in the soil of sea buckthorn forests in the water-wind erosion crisscross region in northern Shaanxi. Soil-related factors such as EC, AP, AK, and TN and meteorological factors such as AAMaxT and AAT were the main drivers of structural changes in the dominant bacterial communities, whereas AK, SOC, AN, Max-WS, and AAT were the main drivers of structural changes in the dominant fungal communities. Compared with meteorological factors, soil-related factors had a greater impact on microbial communities. This study offers valuable insights into the diversity of soil microbial communities in the water-wind erosion crisscross region and the mechanisms underlying the interaction between them and environmental factors.

## Data availability statement

The datasets presented in this study can be found in online repositories. The data presented in the study are deposited in the NCBI repository, accession number was PRJNA912882. The web site of the data repository is https://www.ncbi.nlm.nih.gov/bioproject/912882.

## Author contributions

Z-YZ: investigation, methodology, data curation, validation, and writing—original draft. F-FQ: supervision, methodology, and writing—original draft. G-QL, C-HL, and NA: conceptualization, data curation, and writing—review and editing. All authors contributed to the article and approved the submitted version.

## Funding

This work was funded by the Shaanxi Natural Science Basic Research Project (2021JQ626), National Natural Science Foundation of China (U2243601, 32060297), China Institute of Water Resources and Hydropower Research (IWHR) R&D Support Programs (SC0145B012022 and SC0202A012018), and Ordos Key Water Conservancy Science and Technology Project (2022-2130310-XX).

## Conflict of interest

The authors declare that the research was conducted in the absence of any commercial or financial relationships that could be construed as a potential conflict of interest.

## Publisher’s note

All claims expressed in this article are solely those of the authors and do not necessarily represent those of their affiliated organizations, or those of the publisher, the editors and the reviewers. Any product that may be evaluated in this article, or claim that may be made by its manufacturer, is not guaranteed or endorsed by the publisher.

## Footnotes

^1^ http://www.personalbio.cn/

^2^ http://data.cma.cn/
